# An Activation-Specific Anti-Mac-1 Designed-Ankyrin-Repeat-Protein Attenuates Colitis in Mice

**DOI:** 10.3390/life13071464

**Published:** 2023-06-28

**Authors:** Istvan Bojti, Qianqi Wang, Tibor Bojti, Felicitas Bojti, Patrick Malcolm Siegel, Timo Heidt, Martin Moser, Christoph Bode, Dirk Westermann, Karlheinz Peter, Philipp Diehl

**Affiliations:** 1 Department of Cardiology and Angiology, University Heart Center Freiburg—Bad Krozingen, Faculty of Medicine, University of Freiburg, Hugstetter Street 55, 79106 Freiburg, Germany; 2 Atherothrombosis and Vascular Biology Laboratory, Baker Heart and Diabetes Institute, Melbourne, VIC 3004, Australia; 3 Department of Medicine, Central Clinical School, Monash University, Melbourne, VIC 3004, Australia; 4 Department of Cardiometabolic Health, University of Melbourne, Melbourne, VIC 3004, Australia

**Keywords:** inflammatory bowel diseases, colitis, DARPin, Mac-1, therapy, DSS

## Abstract

(1) Background: Inflammatory bowel diseases are complex and multifactorial disorders of unknown etiology. The extravasation of activated leukocytes is a critical step in the pathogenesis of these diseases. Leukocyte integrin Mac-1 (α_M_β_2_; CD11b/CD18) is crucial for the extravasation of myeloid cells, and a novel activation-specific anti-Mac-1 Designed Ankyrin Repeat protein (DARPin F7) is a promising therapeutic agent for inflammatory diseases. In its activated conformation, Mac-1 expresses the high-affinity binding site I-domain, which the DARPin F7 selectively targets. In our study, we aimed to explore the therapeutic potential of anti-Mac-1 DARPin F7 in murine dextrane sodium sulfate (DSS)-induced colitis. (2) Methods: C57BL/6J mice received 3% DSS drinking water for five days, followed by normal drinking water for one week. The mice were treated with DARPin F7 or a control substance daily via intraperitoneal injections. Disease activity index (DAI), colon length, myeloperoxidase (MPO) activity measurements, H&E staining, and qRT-PCR were conducted after euthanizing the mice on day 12. (3) Results: Treatment with DARPin F7 resulted in less pronounced colon shortening and significantly lower histological scores. The DARPin F7-treated animals experienced substantially less disease and myeloperoxidase (MPO) activity. Animals that received DARPin F7 treatment suffered less weight loss and recovered from the weight loss more efficiently. Treatment with DARPin F7 also led to significantly reduced mRNA expression of inflammatory cytokines. (4) Conclusion: Anti-Mac-1 treatment markedly reduced disease activity and inflammatory reaction accompanying DSS-induced colitis in mice.

## 1. Introduction

Inflammatory bowel disease affects the small intestine and the colon and is characterised by a dysregulated immune response [1]. The principal types of IBDs, Crohn’s disease (CD) and ulcerative colitis (UC), often occur at a young age and are accompanied by physical stress and severe complications, requiring lifelong therapy [2]. The impaired quality of life, therapy costs, and rising incidence of IBDs are all contributing to an ever-growing pressure on health care systems [3]. IBD is the result of an exaggerated immune response in genetically susceptible patients with a dysregulation of the innate and adaptive immune systems, causing robust neutrophil accumulation and mucosal injury [1]. Our current knowledge of IBD pathogenesis relies primarily on the exploration of multiple cell trafficking pathways controlling the physiologic and pathological inflammatory response. Targeted interference with immune cell trafficking is a promising option to dampen the exaggerated immune response against translocated luminal antigens [4].The exact etiology of the disease is not entirely clear yet, but environmental factors, such as antibiotics, dietary components, or microbial exposure are known to be important disease initiators or reactivators [4]. Not only genetic and environmental factors, but novel therapeutics in oncology, such as immune checkpoint inhibitors (ICI), are also major risk factors of autoimmune colitis, which can reach therapy-limiting severity [5]. 

The most severe of the immune checkpoint inhibitor-related adverse events is the immune checkpoint inhibitor-colitis, with the most cases of therapy discontinuation and fatalities [6].

Currently, IBD therapy is limited to classical anti-inflammatory drugs (local treatment with 5-Aminosalicylates or systemically treated with corticosteroids), classic immunosuppressive drugs (azathioprine or 6-mercaptopurine, methotrexate, and ciclosporin-A or tacrolimus), and biological therapy (anti-TNFα agents) [7]. In the last two decades, anti-integrin antibodies, such as natalizumab or vedolizumab, have been developed that specifically interfere with T-cell homing through the endothelium, with less serious side effects and a better effectivity by the latter medicament [8]. As approximately 30–50% of patients do not respond to anti-TNFα agents, which are the most potent of the therapies mentioned above, there is an urgent need for novel therapeutic agents. A further limitation of actual therapeutic agents is their potential life-threatening immunosuppressive effect. 

Integrins play a key role in cell adhesion, migration, activation, and communication [9] and are intriguing targets for drug development. With beneficial effects in ulcerative colitis and Crohn’s disease, the anti-α_4_β_7_ integrin antibodies natalizumab and vedolizumab are good examples of the potential of anti-integrin therapy in inflammatory diseases [4]. However, the unspecific, generalised anti-inflammatory and other side effects of anti-integrin therapy have also resulted in multiple drug development failures [10,11]. 

Meanwhile T and B-cell homing is controlled in a similar way, thus the α_4_β_7_ integrin antibodies inhibit both cell types’ migration, innate immune cells use alternative pathways for homing into target organs [12]. Selectins and ICAM-1-interacting integrins are responsible for regulating the adhesion of monocytes and neutrophils to the endothelium. The β_2_ integrins are an integrin family that mediates ICAM-1 binding. These integrins share a common chain—CD18—with a promiscuous ligand binding affinity [13]. Efforts to inhibit the ICAM-1–β_2_ integrin interaction in IBDs have failed in clinical studies despite the beneficial effects reported in preclinical studies [14].

Mac-1 is a β_2_ integrin expressed mainly by polymorphonuclear cells, monocytes, macrophages, and natural killer cells. Mac-1 is a promising therapeutic target, as it is important for leukocyte adhesion and migration to and through the endothelium [15]. The leukocyte adhesion cascade is a well-designed, redundant system, which enables the leukocytes to use more than one receptor for distinct but crucial processes such as capture, rolling, crawling, and arrest. This redundancy enables interference with only selected targets meanwhile the immune system is further capable of defending itself against invading microorganisms or external/internal stress factors [16]. Mac-1 contains an alpha (CD11b) and beta (CD18) subunit and forms a non-covalently associated dimer. CD11b contains an I-domain, the main binding site for its ligands [17]. This high-affinity binding site becomes exposed upon activation, driven by a conformation change of the integrin [18]. After exposing the high affinity binding site, Mac-1 fulfils its main function by strengthening the binding between the rolling leukocytes and endothelium on the site of inflammation. Mac-1 is responsible not only for the firm adhesion but also for crawling to preferred hotspots where the transmigration occurs [19].

By targeting the I-domain of Mac-1, the Designed Ankyrin Repeat Protein (DARPin) F7 selectively binds to the activated conformation of Mac-1, and therefore inhibits only the activated leukocytes. This leads to an improved side-effect profile without severe immunosuppression [20]. DARPins are designed binding molecules derived from naturally occurring ankyrin repeat proteins. They either target selected molecules, such as the I-domain of Mac-1 (F7), or they are designed to not bind to any known epitopes (control DARPin) [21]. DARPins are capable of withstanding a wide range of temperatures and pH values, thus making them robust binding molecules. Their production using a bacterial expression system and straightforward purification, even on a large scale, are further advances compared to other antibody drugs or small binding molecules. These factors make DARPins a low-cost and ideal therapeutic agent. A further advantage of DARPins compared to commercially available antibodies are their small size (≈14–21 kD), which enables more effective tissue penetration, even in inflamed tissues [20]. 

In this study, we aimed to investigate the potential therapeutic effects of DARPin F7 in a murine DSS-induced colitis model.

After successful induction of the DSS-colitis, we observed a significantly reduced inflammatory response in animals treated with DAPRin F7 compared to the control substances. We also found significantly reduced histological signs of colonic inflammation. The DAPRin F7-treated animals experienced a reduced disease activity. We identified DARPin F7 as a potential candidate for further research in the field of colonic inflammation.

## 2. Materials and Methods

### 2.1. Designed Ankyrin Repeat Proteins (DARPins)

Activation-specific Mac-1 binding (F7) and non-binding control (E3_5) DARPin production and purification were performed as previously described [20]. 

### 2.2. Activation Specific DARPin Binding

DARPin functionality was determined using flow cytometry. We assessed the DARPin binding in whole mouse blood. A total of 100 µL of freshly obtained heparinized blood was stimulated using Phorbol-12-myristat-13-acetat (PMA) or PBS + Ca^2+^ and Mg^2+^ as a negative control. After red blood cell lysis and centrifugation, the cells were resuspended in 100 µL PBS + Ca^2+^ and Mg^2+^ + 0.1% BSA. After binding with DARPins (F7 and E3_5) for 15 min on ice, mouse leukocytes were stained with CD45 (BV510), CD3 (PerCP-Cy5-5), CD19 (PE-Cy7), CD11b, (PE-Texas Red), Ly6G (PE), Ly6C (V450), and anti-His (AF488).

### 2.3. DSS-Induced Colitis

Female C57BL/6J mice aged 8–12 weeks were purchased from Janvier Labs (Le Genest-Saint-Isle, France). The animals were housed in the Center for Experimental Models and Transgenic Service, Freiburg, Germany. All experiments were conducted strictly in line with German animal protection laws and regulations based on the Directive 2010/63/EU of the European Parliament [22], and also in accordance with good animal practice, as defined by the Federation of Laboratory Animal Science Associations and the national animal welfare body GV-SOLAS. The experimental protocols were approved by the responsible regional authorities (‘Tierversuchskomission Regierungspräsidium Freiburg” 35-9185.81/G-20/07).

The mice received 3% DSS in their drinking water for 5 days, followed by 7 days of normal drink water. The healthy control mice had free access to drinking water. The DSS solutions were prepared fresh daily. The number of mice in each treatment group was as follows: DARPin F7—*n* = 10, control DARPin—*n* = 10, PBS—*n* = 5. Additionally, there were 5 healthy control animals. The treatment groups received 0.2 mL (1 mg/mL) of DARPin F7, control DARPin intraperitoneally, or 0.2 mL of phosphate buffered saline as control substance on a daily basis.

After euthanasia by cervical dislocation, the colon was dissected and cut in half longitudinally. One half was rolled up in a Swiss roll for histological analysis, and the other half was homogenized for qRT-PCR and the MPO activity assay.

### 2.4. Quantitative Real-Time Polymerase Chain Reaction (qRT-PCR)

The RNA was extracted from homogenized colonic tissue using Qiazol and an RNeasy Mini Kit (Qiagen, Valencia, CA, USA). Quantitative TaqMan PCR were performed as described elsewhere [23]. The following TaqMan probes were used: Mm00446190_m1 (IL6), Mm01288386_m1 (IL10), Mm00434228_m1 (IL1b), and Mm01168134_m1 (IFN gamma). The data were analysed using the 2^–∆∆Ct^ method. The mRNA levels were normalized using GAPDH as a housekeeping gene, then standardized to the mean value of the respective healthy control group.

### 2.5. MPO Activity Assay

An MPO activity assay was performed following the manufacturer’s (Sigma Aldrich, St. Louis, MO, USA, ab105136) instructions. The colonic tissue was homogenized and centrifuged. The protein concentration of the supernatant was determined using a commercially available BCA assay. The MPO activity was normalized to the protein concentration. One unit of MPO represents the amount of MPO which hydrolyses the substrate and generates taurine chloramine to consume 1.0 μmol of TNB per minute at 25 °C. The MPO activity was divided by the protein amount measured in the lysed colon tissue.

### 2.6. Histology

The longitudinally half-cut colon was rolled in a Swiss roll and embedded in OCT compound. The fast-frozen tissue blocks were cut in 8 µm-thick sections, and hematoxylin and eosin staining was performed according to standard protocols. Histological scoring was performed in a blinded manner based on the scoring system of Kitajima et al. [24] (Table 1). 

### 2.7. Disease Activity Index

The disease activity index was calculated from the combined score of weight loss, stool consistency, and rectal bleeding divided by 3 (Table 2). The weight loss was calculated every day by comparing the actual weight to the starting point. 

### 2.8. Statistics

The data are presented as the mean ± standard error of the mean (SEM). A Kolmogorov–Smirnov test was performed to test for normal or lognormal distribution. Data with lognormal distribution were log-transformed. A one-way ANOVA with Fisher’s Least Significant Difference test was used when the means were compared. The statistics were performed using GraphPad Prism V9.0.1 software (GraphPad, San Diego, CA, USA). A *p*-value ≤ 0.05 was considered statistically significant. * *p* < 0.05, ** *p* < 0.01, *** *p* < 0.001. 

## 3. Results

### 3.1. Body Weight and Colon Length

**Figure 1:**
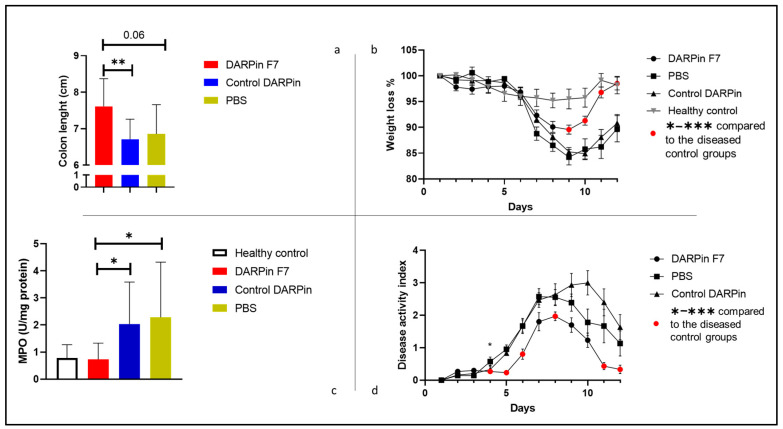
Colon length, body weight courses, disease activity score, and MPO activity. (**a**) DARPin F7 treatment was protective against colon shrinkage. Colon lengths are presented as mean ± SEM: DARPinF7: 7.6 ± 0.24, control DARPin: 6.7 ± 0.55, PBS: 6.8 ± 0.67 (**b**) Animals treated with DARPin F7 had ameliorated weight loss compared to both control groups. The red marks on (**b**) indicate significant differences compared to both control groups. (**c**) DARPin F7 therapy significantly reduced the MPO activity compared to both control groups. (**d**) DARPin F7 treatment reduced disease activity, delayed the onset of symptoms, and enabled a full recovery. The red marks on Figure 1d indicate significant differences compared to both control groups. Healthy control, *n* = 5; PBS, *n* = 5; DARPin F7, *n* = 10; control DARPin, *n* = 10. Female C57BL/6J mice aged 8–12 weeks old received 3% DSS in their drinking water for 5 days, followed by 7 days of normal drinking water. Healthy control mice had free access to drinking water. Abbreviations: DARPin: Designed Ankyrin Repeat Proteins, PBS: phosphate buffered saline, DSS: dextrane sodium sulfate, * *p* < 0.05, ** *p* < 0.01.

We observed significant weight loss in all groups treated with DSS. As shown in Figure 1 and supplementary Table S1, the weight loss levels in the group treated with DARPin F7 were significantly lower compared to the control groups. Moreover, the animals in the F7 group recovered faster and reached the weight of healthy control animals by day 12. The colon length measurement revealed a major difference between the groups treated with F7 and the control DARPin. There was a marked (but not significant, *p* = 0.06) difference between the groups treated with F7 and phosphate buffered saline (PBS).

### 3.2. Disease Activity Score

The disease activity score was calculated as described in the Materials and Methods section. DAPRin F7 therapy not only delayed the time point of symptom onset, but also reduced the maximal activity of the disease. The fast and full recovery in the group treated with DARPin F7 was also visible based on the disease activity score (see Figure 1d and Supplementary Table S2). 

### 3.3. MPO Activity Assay

After the measurement of the protein content in the supernatant of the lysed colon tissue, we were able to normalise the MPO activity measured in the samples. As shown in Figure 1c, we measured significantly reduced MPO activity in the mice treated with DARPin F7 compared to both control groups.

### 3.4. Histological Score

**Figure 2:**
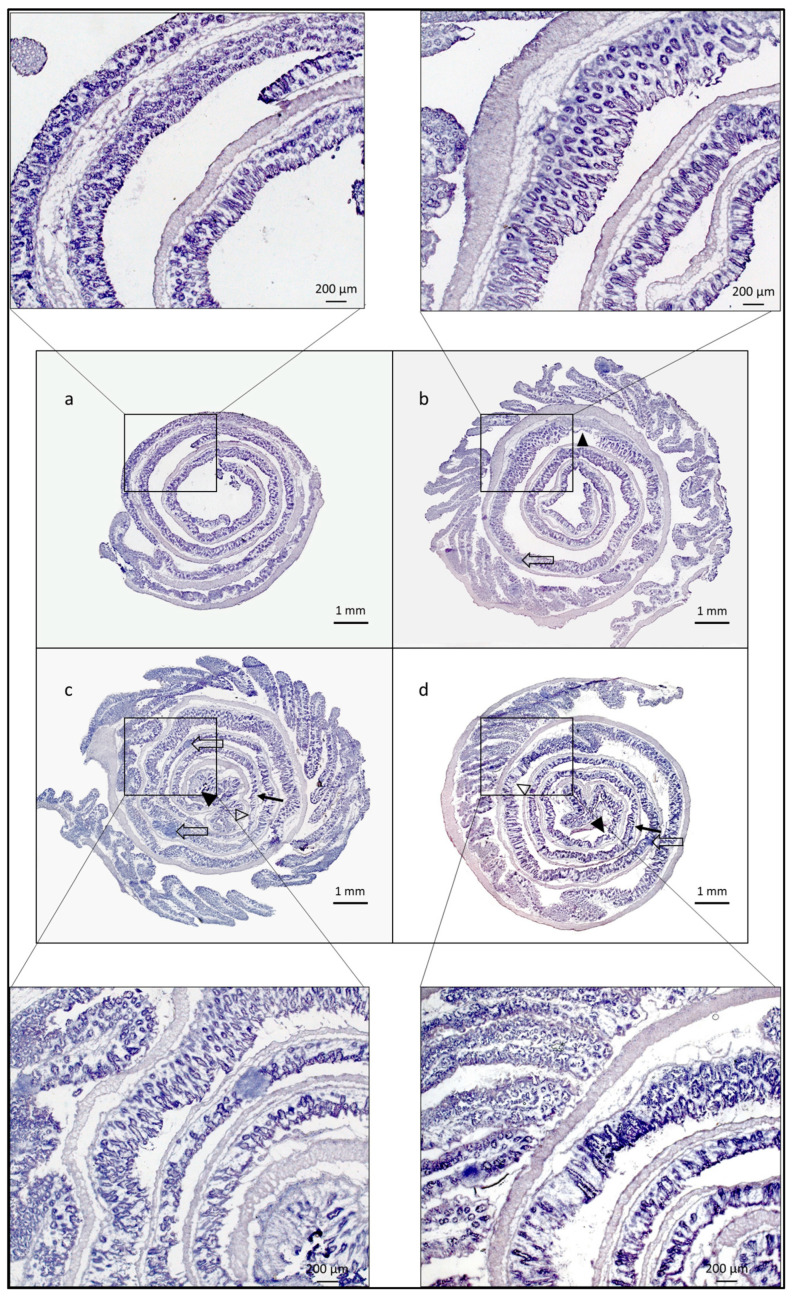
Representative histological images. DARPin F7 therapy dampened epithelium and crypt damage, while also limiting the extension of the lesions. (**a**) Healthy control, (**b**) DARPin F7, (**c**) control DARPin, (**d**) PBS. Black arrowheads: ulceration, transparent arrowheads: epithelial damage, black arrow: loss of the basal crypts, transparent arrows: lymphoid conglomerates. H/E, 50× magnification. Healthy control, *n* = 5; PBS, *n* = 5; DARPinF7, *n* = 10; control DARPin, *n* = 10. Female C57BL/6J mice aged 8–12 weeks old received 3% DSS in their drinking water for 5 days, followed by 7 days of normal drinking water. Healthy control mice had free access to drinking water. Abbreviations: DARPin: Designed Ankyrin Repeat Proteins, PBS: phosphate buffered saline, DSS: dextrane sodium sulfate.

**Figure 3:**
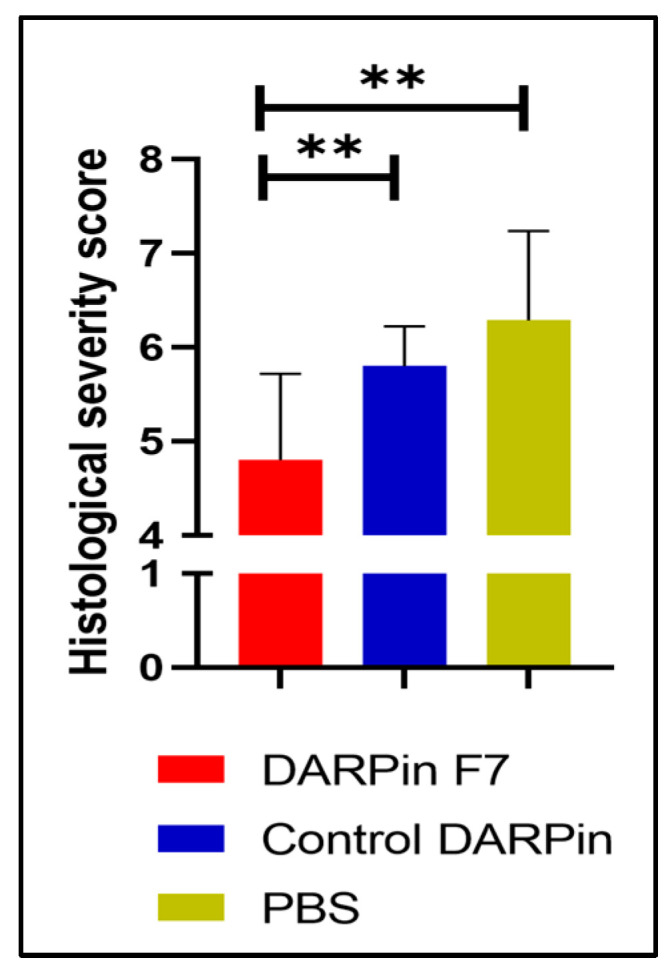
Histological severity score. Histological scoring was performed in a blinded manner based on the scoring system of Kitajima et al. [24]. Results are presented as mean ± SEM. DARPin F7 vs. control DARPin: 4.8 (0.29) vs. 5.8 (0.13), *p* = 0.005; DARPin F7 vs. PBS: 4.8 (0.29) vs. 6.0 (0.32), *p* = 0.006. PBS, *n* = 5; DARPinF7, *n* = 10; control DARPin, *n* = 10. Female C57BL/6J mice aged 8–12 weeks old received 3% DSS in their drinking water for 5 days, followed by 7 days of normal drinking water. Abbreviations: DARPin: Designed Ankyrin Repeat Proteins, PBS: phosphate buffered saline, DSS: dextrane sodium sulfate, ** *p* < 0.01.

We observed typical alterations in colonic inflammation through histological analysis, as described by Kitajima et al. [24]. The DARPin F7 therapy reduced the damage to crypts and epithelial cells themselves, while also preventing the extension of the damage. Representative histological images are included in Figure 2 and the histological severity score in Figure 3.

### 3.5. Cytokine RNA Analysis in Colonic Tissue

**Figure 4:**
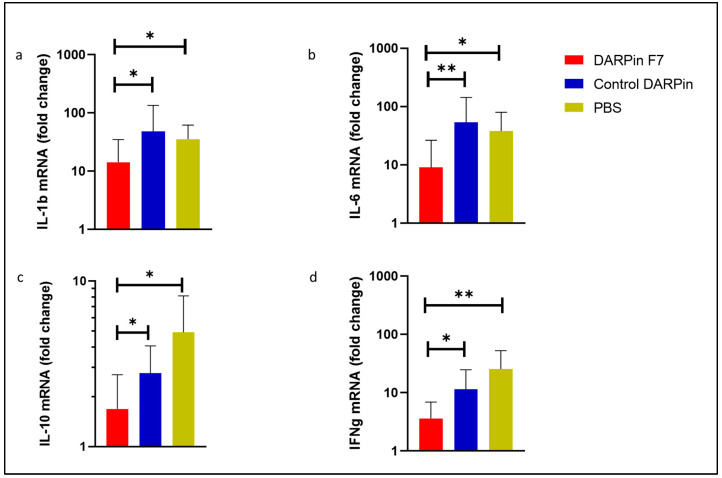
Relative mRNA expression of the measured cytokines compared to healthy control animals in homogenised colon tissue. DARPin F7 therapy reduced the expression of proinflammatory (**a**) IL-1β, (**b**) IL-6, (**c**) anti-inflammatory IL-10, and (**d**) IFNγ cytokines’ mRNA. Healthy control, *n* = 5; PBS, *n* = 5; DARPinF7, *n* = 10; control DARPin, *n* = 10. Female C57BL/6J mice aged 8–12 weeks old received 3% DSS in their drinking water for 5 days, followed by 7 days of normal drinking water. Healthy control mice had free access to drinking water. Abbreviations: DARPin: Designed Ankyrin Repeat Proteins, PBS: phosphate buffered saline, DSS: dextrane sodium sulfate, * *p* < 0.05, ** *p* < 0.01.

The cytokine analysis revealed significant differences in the expression of measured mRNAs. Pro-inflammatory cytokines IL-1b, IL-6, IFNγ, and the anti-inflammatory cytokine IL-10 were all significantly elevated in the control groups compared to the animals treated with DARPin F7 as seen on Figure 4.

### 3.6. DARPin Functionality

CD11b-positive cells such as neutrophils (CD45^+^CD11b^+^Ly6C^+^Ly6G^+^) and monocytes (CD45^+^CD11b^+^Ly6C^+^Ly6G^−^) showed an activation-specific enhancement of the mean fluorescent intensity for AF488, signaling an elevated binding of the DARPin F7 to the activated CD11b after PMA activation compared to the non-activated cells or the negative control DARPin E3_5 (Supplementary Figure S1).

## 4. Discussion

In this study, we demonstrated the safety and effectiveness of DARPin F7 treatment in acute DSS colitis in mice. The mice treated with the DARPin F7 experienced reduced colonic inflammation and weight loss as well as a faster recovery. In our descriptive analysis, we identified DARPin F7 as a potential candidate for further research on the ever-growing field of inflammatory bowel diseases and ICI-related colitis.

The unsatisfactory therapeutical options currently available for IBDs and the ever-growing population of cancer patients who receive immune checkpoint inhibitors are drawing attention to the need for novel drugs to treat intestinal inflammation. An ideal agent would be able to reduce intestinal inflammation without impairing host defence, and to facilitate the continuation of immune checkpoint inhibitor therapy. Anti-integrin drugs are established candidates in this field, and the inhibition of α_4_β_7_ integrin has already proven safe and effective in selected IBD patients. Nevertheless, there is still room for improvement in the available therapy options [26]. Inhibition of β_2_-integrin function is a promising therapeutic candidate, as the expression of these integrins is significantly up-regulated in patients with ulcerative colitis or Crohn’s disease [27]. 

There is a significant histological and endoscopic overlap between IBDs and ICI-induced colonic inflammation, with more pronounced neutrophil infiltration in ICI therapy [5,6,28]. Therapeutic response to corticosteroids and anti-TNF alpha antibody is also similar in these disease entities [29].

In order to investigate the effects of DARPin F7 on acute DSS colitis in mice, we chose a validated, reproducible, and robust method to induce the disease [30]. The DSS colitis model is characterized by overwhelming neutrophil infiltration, thus making it an ideal model to demonstrate the therapeutic potential of activation-specific Mac-1 inhibition. Furthermore, there is clear evidence for the crucial role of ICAM-1 in the pathogenesis of DSS colitis [31]. Thus, the previously demonstrated potent inhibition of ICAM-1–Mac-1 interaction by DARPin F7 suggests a beneficial effect in DSS colitis [20]. 

We successfully produced and tested the activation-specific binding of the DARPin F7 on mouse leukocytes. The non-binding property of the control DARPin was also assessed using flow cytometry. This analysis is important to demonstrate cell and activation-specific binding of our DARPin.

We successfully induced acute DSS colitis with similar disease activity, weight loss, and histological changes to previous studies [30,32,33]. Our findings support the hypothesis that neutrophils indeed play a crucial role in the pathogenesis of DSS-induced colitis, and the inhibition of their homing results in less severe intestinal inflammation, accompanied by lower disease activity and a faster resolution of the colitis in the acute phase. 

It is well-known that neutrophils play an important role in intestinal homeostasis by co-orchestrating antimicrobial defence and mucosal wound healing. However, their interaction with intestinal recruitment can be both beneficial and detrimental, making them a double-edged sword [34]. The prominent infiltration of neutrophils into colonic tissue is a hallmark of inflammatory bowel disease and ICI-related colitis. Their exaggerated presence and activation lead to a cascade of tissue-damaging processes [35]. Neutrophils can produce MPO, which is an important regulator of inflammatory processes. MPO is not only a highly cytotoxic agent that is released primarily by activated neutrophils but is also a potent suppressor of neutrophil apoptosis. This effect relies on the Mac-1–MPO interaction [36]. To prove the presence of functional neutrophils in colon tissue, we investigated the MPO activity in the supernatant of the lysated colon, as this enzyme is a hallmark of neutrophil-driven inflammation [37]. In line with others, we found significantly elevated MPO activity in the control groups, signalising a more pronounced neutrophil infiltration [38]. Lower MPO activity is not only a footprint of dampened inflammation, but also a therapeutic goal. As Lau et al. demonstrated, elevated MPO levels indicate an inflammatory state, but more importantly, MPO interacts with Mac-1 to further activate the inflammatory process [39]. Thus, the activation-specific inhibition of Mac-1 may not only demonstrate its beneficial effects by suppressing leukocyte recruitment, but also by preventing the MPO–Mac-1 interaction.

The findings of Abdelbaqi et al. highlight the importance of intact Mac-1 in colitis, as they demonstrated that CD11b-deficient mice display enhanced disease activity [32]. In our current and previous experiments, we did not see increased distress or any other signs of infection under continuous DARPin F7 treatment [23]. This observation highlights the importance of targeted CD11b inhibition.

Using a DARPin targeted against a β_2_ integrin may seem counterproductive in the case of the inflammatory bowel disease, as β_2_ integrins play a crucial role in host defence against bacterial infections [40]. Concerns regarding enhanced bacterial translocation and elevated disease activity could be addressed by observations in genetically modified mice that lack specific β_2_-integrin subunits. In the absence of CD11b, CD11a, or CD18, there was no sign of elevated bacterial translocation [32]. Our previous findings using cecal ligation and puncture polymicrobial sepsis models are in line with these observations, as we also did not observe an elevated inflammatory response under DARPinF7 therapy [20].

The inhibition of neutrophil binding and transmigration without obvious impairment to the host defence may be due to the specific binding of the tested DARPin F7. It preferentially binds to the I-domain in an activated form of Mac-1 without interacting with other binding sites, such as the fourth blade of the β-propeller in CD11b or the lectin site, which are important for host defence (e.g., phagocytosis) [17]. The effects of long-term DARPin F7 therapy on the immune system are yet to be elucidated and are topic for future experiments.

Apart from the involvement of neutrophils, there are other shared characteristics of DSS and ICI-related colitis, such as the elevation of IL-1β or IL-6 [28,41]. In our model, we reproduced a relevant elevation of both cytokines’ mRNA expression, with a significant reduction in the DARPin F7-treated group. Therapies aimed at reducing elevated IL-1β and IL-6 levels or receptor binding are promising options for fighting ICI-toxicities and autoimmune colitis [4,42].

IL-10 has a confirmed strong anti-inflammatory effect and plays an important role in maintaining gut homeostasis [43,44]. The therapeutic potential and biology of this cytokine, particularly in relation to autoimmunity, has been demonstrated in various human and animal diseases [45]. Interestingly, its elevation is associated with adverse outcomes under ICI therapy, signalling an elevated risk for autoimmune complications, such as pneumonitis or colitis [46,47]. In contrast, as discussed by Moran et al., reduced IL-10 or IL10R expression is associated with early onset IBD [48]. We observed significantly lower IL-10 expression levels under DARPin F7 therapy compared to the control groups. This discrepancy could be linked to the time course of IL-10 secretion, with peak levels occurring 7–14 days after the initiation of various experimental autoimmune diseases [49,50]. Lower IL-10 levels under DARPin F7 therapy are likely due to a dampened autoimmune response, leading to a lower and earlier peak. Elevated IL-6 levels in control groups are followed by a more active inflammation, and a higher IL-6-dependent induction of IL-10 production in order to control the inflammation could contribute to this observation [51]. As an alternative explanation, the blocking of Mac-1 can reduce IL-10 expression, as demonstrated by Hu et al. [52]. 

IBDs are characterised not only by disruption of the intestinal epithelial barrier, but also by dysfunction of the endothelial vascular barrier [53]. IFN-γ plays a key role in this process, and it is one of the most elevated cytokines in IBDs [54]. We observed relevant elevations of IFN-γ mRNA expression in both control groups, and a significantly lower level under DARPin F7 treatment. Experiments with IFN-γ knockout mice demonstrated an indispensable role of IFN-γ in the pathogenesis of DSS colitis in mice; thus, reducing its levels is an important objective in the treatment of IBDs [55]. 

Our study has some limitations. As with every animal model, the DSS-induced colitis model mimics the human disease, but it does not replicate it with 100% accuracy. Mac-1 is also found on B-cells and may attenuate DSS-induced colitis through this cell population, which we did not investigate in our study. This issue should be explored in future experiments [56]. The direct enhancement of IL-10 expression by Mac-1 and the potential interaction of DARPin F7 through this pathway is an equally important aspect to investigate. Future experiments should also focus on expression changes on the protein level. We focused on the acute phase of the disease; therefore, we did not investigate the effects of DARPin F7 therapy in the chronic phase of the disease. The observed differences between the control groups did not reach statistical significance on any occasion, and we considered them to be random fluctuations. This observation may rely on the number of treated animals.

## 5. Conclusions

The activation-specific inhibition of the I domain on the Mac-1 integrin by DARPin F7 is highly effective and safe in a DSS-induced mouse model of colitis. The DARPin F7 therapy attenuated colonic inflammatory reactions, resulting in less disease activity and faster disease resolution. Further preclinical studies are warranted to ultimately advance DARPin F7 toward clinical translation. Nevertheless, the current results hold promise for a new DARPin-based, activation-specific Mac-1 inhibition as a novel therapy for colitis and other inflammatory diseases. 

## Figures and Tables

**Table 1:** Scoring system according to Kitajima et al.

	Score	
Damage	0	None
	1	loss of the basal 1/3 of crypt
	2	loss of the basal 2/3 of crypt
	3	loss of the entire crypt, but intact surface epithelial cells
	4	loss of the both the entire crypt and the surface epithelial cells (erosion)
Extension	0	None
	1	Focal
	2	lesion involving 1/3 intestine
	3	lesion involving 2/3 intestine
	4	lesion involving the entire intestine

**Table 2:** Criteria for scoring the disease activity index (DAI), adapted from Rath et al. [25].

Score	Weight Loss (%)	Stool Consistency	Rectal Bleeding
0	None	Normal (well formed pellets)	Negative
1	1–5		
2	6–10	Loose (pasty stool that does not stick to the anus)	Gross bleeding
3	11–15		Gross bleeding > 1 d
4	>15	Diarrhea (liquid stool that sticks to the anus)	Gross bleeding > 2 d

## Data Availability

The datasets generated and analyzed in this study are available from the corresponding author upon reasonable request.

## Supplementary Materials

The following supporting information can be downloaded at: https://www.mdpi.com/article/10.3390/life13071464/s1, Supplementary Figure S1: There was a significant growth of the mean Alexa fluor 488 fluorescent intensity (MFI) after Phorbol-12-myristat-13-acetat (PMA) stimulation in the examined cell populations. Mean MFI growth % in B-cells, T-cells, monocytes and neutrophils respectively (DARPin F7 vs. Control DARPin): −22 (SD: 3.6) vs. −10.5 (SD: 23.3), 7.3 (SD: 3) vs. −15 (SD: 19), 86.8 (SD: 39.3) vs. −9.6 (SD: 14.7), 241 (SD: 80) vs. −2.5 (SD: 6.3). 100 µL freshly obtained heparinized blood was stimulated with PMA or PBS + Ca^2+^ and Mg^2+^ as negative control. After red blood cell lysis and centrifugation cells were resuspended in 100 µL PBS + Ca^2+^ and Mg^2+^ + 0.1% BSA. After binding with DARPins (F7 and E3_5) for 15 min on ice, mouse leukocytes were stained with CD45 (BV510), CD3 (PerCP-Cy5-5), CD19 (PE-Cy7), CD11b, (PE-Texas Red), Ly6G (PE), Ly6C (V450) and anti-His (AF488). B-cells: CD45+CD19+, T-cells: CD45+CD3+, Monocytes: CD45+CD11b+Ly6C+Ly6C-, Neutrophils: CD45+CD11b+Ly6C+Ly6C+. Abbreviations: MFI: mean fluorescent intensity, PMA: Phorbol-12-myristat-13-acetat, DARPin: Designed Ankyrin Repeat Proteins, PBS: phosphate buffered saline; Supplementary Table S1: Weight loss comparison of the three treatment groups. Pairwise comparisons were performed on each day of the experiment. A two-tailed student’s *t*-test was used when means of two groups were compared. Abbreviations: DARPin: Designed Ankyrin Repeat Proteins, SD: standard deviation.; Supplementary Table S2: Disease activity score of the three treatment groups. Pairwise comparisons were performed on each day of the experiment. A two-tailed student’s *t*-test was used when means of two groups were compared. Abbreviations: DARPin: Designed Ankyrin Repeat Proteins, SD: standard deviation.
