# Supplementary material for: An Activation-Specific Anti-Mac-1 Designed-Ankyrin-Repeat-Protein Attenuates Colitis in Mice
Source: Life (Basel). 2023 Jun 28;13(7):1464. doi: 10.3390/life13071464 (PMC10381548; doi:10.3390/life13071464)
Supplement: Supplementary file 1 [file life-13-01464-s001.zip › life-2274701-supplementary.pdf]

An Activation-Specific Anti-Mac-1 Designed-Ankyrin-Repeat-Protein Attenuates Colitis in Mice

Istvan Bojti [1,*], Qianqi Wang [1], Tibor Bojti [1], Felicitas Bojti [1], Patrick Malcolm Siegel [1], Timo Heidt [1], Martin Moser [1], Christoph Bode [1], Dirk Westermann [1], Karlheinz Peter [2,3,4] and Philipp Diehl [1]

[1] Department of Cardiology and Angiology, University Heart Center Freiburg—Bad Krozingen, Faculty of Medicine, University of Freiburg, Hugstetter Street 55, 79106 Freiburg, Germany
[2] Atherothrombosis and Vascular Biology Laboratory, Baker Heart and Diabetes Institute, Melbourne, VIC 3004, Australia
[3] Department of Medicine, Central Clinical School, Monash University, Melbourne, VIC 3004, Australia
[4] Department of Cardiometabolic Health, University of Melbourne, Melbourne, VIC 3004, Australia
* Correspondence: istvan.bojti@uniklinik-freiburg.de; Tel: +49-761/270-34010

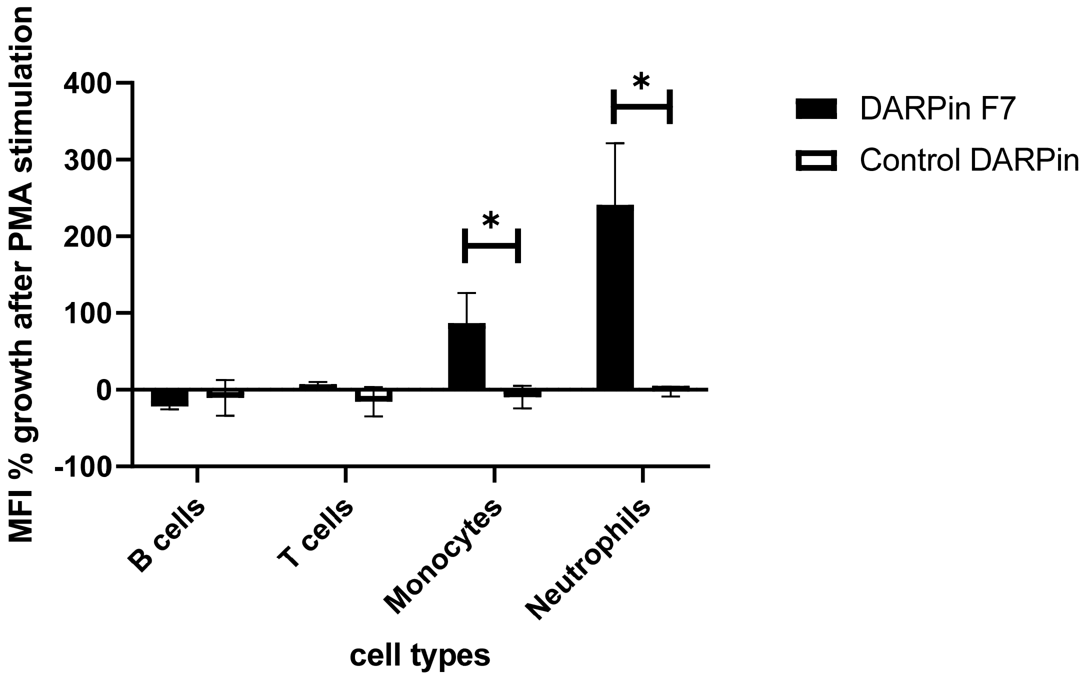

Supplementary Figure S1: There was a significant growth of the mean Alexa fluor 488 fluorescent intensity (MFI) after Phorbol-12-myristat-13-acetat (PMA) stimulation in the examined cell populations. Mean MFI growth % in B-cells, T-cells, monocytes and neutrophils respectively (DARPin F7 vs Control DARPin): -22 (SD: 3.6) vs. -10.5 (SD: 23.3), 7.3 (SD: 3) vs. -15 (SD:19), 86.8 (SD: 39.3) vs. -9.6 (SD: 14.7), 241 (SD: 80) vs. -2.5 (SD: 6.3). 100µl freshly obtained heparinized blood was stimulated with PMA or PBS + Ca^{2+} and Mg^{2+} as negative control. After red blood cell lysis and centrifugation cells were resuspended in 100 µl PBS + Ca^{2+} and Mg^{2+} + 0.1% BSA. After binding with DARPins (F7 and E3_5) for 15 min on ice, mouse leukocytes were stained with CD45 (BV510), CD3 (PerCP-Cy5-5), CD19 (PE-Cy7), CD11b, (PE-Texas Red), Ly6G (PE), Ly6C (V450) and anti-His (AF488). B-cells: CD45+CD19+, T-cells: CD45+CD3+, Monocytes: CD45+CD11b+Ly6C+Ly6C-, Neutrophils: CD45+CD11b+Ly6C+Ly6C+. Abbreviations: MFI: mean fluorescent intensity, PMA: Phorbol-12-myristat-13-acetat, DARPin: Designed Ankyrin Repeat Proteins, PBS: phosphate buffered saline.

Days	DARPin F7		PBS		Control DARPin		p-value	
	Mean	SD	Mean	SD	Mean	SD	DARPinF7 vs. PBS	DARPinF7 vs. Control DARPin
1	100	0	100	0	100	0		
2	97,81	2,27	99,34	1,597	99,21	1,97	0,20	0,16
3	97,42	3,14	100,6	2,489	99,09	0,8277	0,07	0,12
4	97,91	3,64	98,84	2,206	99,06	1,168	0,61	0,35
5	98,01	3,37	99,41	1,123	98,64	1,561	0,39	0,60
6	96,86	3,012	96,54	2,515	96,19	1,772	0,84	0,55
7	92,32	3,38	88,78	2,903	91,52	1,882	0,07	0,52
8	90,1	3,38	86,52	2,63	88,2	2,069	0,06	0,15
9	89,57	2,8	84,22	3,343	85,26	2,669	**0,01**	**<0,01**
10	91,31	2,79	85,75	4,561	84,91	3,263	**0,01**	**<0,01**
11	96,79	3,39	86,22	5,012	88,07	4,851	**<0,01**	**<0,01**
12	98,51	3,98	89,73	5,697	90,83	5,266	**<0,01**	**<0,01**

Supplementary Table S1: Weight loss comparison of the three treatment groups. Pairwise comparisons were performed on each day of the experiment. A two-tailed student's t-test was used when means of two groups were compared. Abbreviations: DARPin: Designed Ankyrin Repeat Proteins, SD: standard deviation.

Days	DARPin F7		PBS		Control DARPin		p-value	
	Mean	SD	Mean	SD	Mean	SD	DARPinF7 vs. PBS	DARPinF7 vs. Control DARPin
1	0,00	0,00	0,00	0,00	0,00	0,00		
2	0,27	0,17	0,14	0,17	0,16	0,17	0,11	0,14
3	0,30	0,18	0,14	0,18	0,20	0,17	0,11	0,23
4	0,27	0,37	0,57	0,37	0,33	0,38	**0,05**	0,64
5	0,23	0,36	0,95	0,36	0,83	0,24	**<0,01**	**<0,01**
6	0,80	0,51	1,67	0,51	1,67	0,74	**<0,01**	**<0,01**
7	1,80	0,66	2,57	0,66	2,47	0,71	0,07	0,08
8	1,97	0,58	2,56	0,58	2,63	1,07	**0,04**	0,08
9	1,70	0,65	2,39	0,65	2,93	1,13	0,07	**<0,01**
10	1,23	1,00	1,78	1,00	3,00	1,19	0,22	**<0,01**
11	0,43	1,16	1,67	1,16	2,40	1,31	**<0,01**	**<0,01**
12	0,33	0,87	1,13	0,87	1,63	1,22	**0,03**	**<0,01**

Supplementary Table S2: Disease activity score of the three treatment groups. Pairwise comparisons were performed on each day of the experiment. A two-tailed student's t-test was used when means of two groups were compared. Abbreviations: DARPin: Designed Ankyrin Repeat Proteins, SD: standard deviation.
